# C. Lockhart Robertson

**Published:** 1897-05-29

**Authors:** 


### The Institutional Workshop.

### OBITUARY.

### C. LOCKHART ROBERTSON, M.D.Cantab., F.R.C.P.Lond.

(By a Correspondent.)

On May 18th there passed away from us for ever one of the most prominent figures of asylum life during the last forty years; and as I saw the grave close over him I felt how inadequate the usual commonplace words of consolation were to those who, like myself, numbered him among their friends. A deeper note of woe had been struck that could not be silenced at once: a heart-felt pang had been received that needed time to assuage. After obtaining his diplomas in Edinburgh, Dr. Robertson joined the army as assistant-surgeon. This work was not wholly congenial to him, and he sent in his papers, subsequently entering at Caius College, Cambridge, and remaining there until he got the M.D., degree. With this he settled in London, and was for a while one of the Queen's Gentlemen-at-Arms. He had, however, always a strong leaning to the specialty of insanity, and in 1859 he abandoned private practice to open the recently-built Sussex County Asylum as medical superintendent. Here all his innate abilities as an organiser found play, and he soon raised the institution to the highest place among asylums of its kind. For many years he, in conjunction with Dr. Maudsley, edited the *Journal of Mental Science*, and it was during these palmy days that it reached the very zenith of its fame, no other period of its existence having approached it in the carefulness of its management or the brilliancy of its contributions. On the death of Sir Charles Hood, Dr. Robertson was appointed Lord Chancellor's Visitor in Lunacy, which post he held until 1896, when he retired on a pension. In his younger days Dr. Robertson was a voluminous writer of articles and pamphlets and abstracts, but these are not much read by the present generation, partly owing to the ephemeral nature of the subjects chosen and partly because his prose, though good, lacked the true classic ring which alone can command a permanent place in letters. A fine German scholar, he has left us as a monument of his patience and industry a translation of Greisinger's work on Mental Diseases. Being a brilliant conversationalist and a widely-read man, there was a peculiar charm in his company. I well remember an evening ten years ago which I spent with him and the late (alas, that I have to write late!) Sir Benjamin Richardson. They had been intimate friends at one time, but had drifted apart, not from any particular reason, but because different walks in life were theirs. A hint from one that he would like to meet the other found effect in a meeting at my club. The dinner was a simple one, for neither was an epicure; one was a teetotaler, the other almost so, but never shall I forget that evening. I sat and listened! One fine thought expressed called forth another. Perhaps an anecdote that had slumbered for years was awakened, and brought others in its train, until the three or four hours flew away and brought to an end one of the most memorable evenings of my life. I believe these old friends never met again, and they have gone into the silent land within a few months of each other. I have often thought Queen Catherine's lines descriptive of Cardinal Wolsley peculiarly applicable to Dr. Robertson:—

> "He was a scholar, and a ripe and good one;
> Exceeding wise, fair spoken and persuading:
> Lofty and sour to them that loved him not;
> But to those men that sought him, sweet as summer."

